# COVID-19 vaccine effectiveness in the paediatric population aged 5–17 years: a multicentre cohort study using electronic health registries in six European countries, 2021 to 2022

**DOI:** 10.2807/1560-7917.ES.2025.30.8.2400450

**Published:** 2025-02-27

**Authors:** Patrícia Soares, Ausenda Machado, Nathalie Nicolay, Susana Monge, Chiara Sacco, Christian Holm Hansen, Hinta Meijerink, Iván Martínez-Baz, Susanne Schmitz, James Humphreys, Massimo Fabiani, Aitziber Echeverria, Ala’a AlKerwi, Anthony Nardone, Alberto Mateo-Urdiales, Jesús Castilla, Esther Kissling, Baltazar Nunes, VEBIS-Lot 4 working group, VEBIS-Lot 4 working group, Diana Lucas, Itziar Casado, Izaak Van Evercooren

**Affiliations:** 1 National Institute of Health Doutor Ricardo Jorge, Lisbon, Portugal; 2 Vaccine Preventable Diseases and Immunisation, European Centre for Disease Prevention and Control (ECDC), Stockholm, Sweden; 3 National Centre of Epidemiology, Institute of Health Carlos III, Madrid, Spain; 4 Centro de Investigación Biomédica en Red (CIBER) on Infectious Diseases, Madrid, Spain; 5 Department of Infectious Diseases, Istituto Superiore di Sanità, Rome, Italy; 6 Department of Infectious Disease Epidemiology and Prevention, Statens Serum Institut, Copenhagen, Denmark; 7 Norwegian Institute of Public Health (NIPH), Oslo, Norway; 8 Instituto de Salud Pública de Navarra – IdiSNA – CIBERESP, Pamplona, Spain; 9 Ministry of Health and Social Security, Directorate of Health, Service epidemiology and statistics, Luxembourg, Luxembourg; 10 Epiconcept, Paris, France; 11 The members of the VEBIS-Lot 4 working group are listed under Collaborators.

**Keywords:** COVID-19 vaccines, paediatrics, electronic health records, vaccine effectiveness

## Abstract

**Background:**

During the first year of the COVID-19 pandemic, vaccination programmes targeted children and adolescents to prevent severe outcomes of SARS-CoV-2 infection.

**Aim:**

To estimate COVID-19 vaccine effectiveness (VE) against hospitalisation due to COVID-19 in the paediatric population, among those with and without previously documented SARS-CoV-2 infection.

**Methods:**

We established a fixed cohort followed for 12 months in Denmark, Norway, Italy, Luxembourg, Navarre (Spain) and Portugal using routine electronic health registries. The study commenced with paediatric COVID-19 vaccination campaign at each site between June 2021 and January 2022. The outcome was hospitalisation with a laboratory-confirmed SARS-CoV-2 infection or COVID-19 as the main diagnosis. Using Cox proportional hazard models, VE was estimated as 1 minus the confounder-adjusted hazard ratio of COVID-19 hospitalisation between vaccinated and unvaccinated. A random-effects meta-analysis was used to pool VE estimates.

**Results:**

We included 4,144,667 5–11-year-olds and 3,861,841 12–17-year-olds. In 12–17-year-olds without previous infection, overall VE was 69% (95% CI: 40 to 84). VE declined with time since vaccination from 77% ≤ 3 months to 48% 180–365 days after immunisation. VE was 94% (95% CI: 90 to 96), 56% (95% CI: 3 to 80) and 41% (95% CI: −14 to 69) in the Delta, Omicron BA.1/BA.2 and BA.4/BA.5 periods, respectively. In 12–17-year-olds with previous infection, one dose VE was 80% (95% CI: 18 to 95). VE estimates were similar for 5–11-year-olds but with lower precision.

**Conclusion:**

Vaccines recommended for 5–17-year-olds provided protection against COVID-19 hospitalisation, regardless of a previously documented infection of SARS-CoV-2, with high levels of protection in the first 3 months of the vaccination.

**Key public health message**

**What did you want to address in this study and why?**

Vaccination programmes were launched in the first year of the pandemic including for children and adolescents to prevent severe outcomes of SARS-CoV-2 infection, despite being rare in younger age groups. We assessed vaccine effectiveness (VE) of COVID-19 vaccines across six European countries in 5–17-years-olds with and without previous SARS-CoV-2 infection. Our 12-month study period allowed examination of VE against Delta and Omicron variants.

**What have we learnt from this study?**

Overall, vaccines recommended for 5–17-year-olds prevented two thirds of COVID-19 hospitalisations, regardless of a previously documented infection of SARS-CoV-2. The level of protection was the highest 3 months following vaccination. Although VE declined over time, vaccinees remained at lower risk of hospitalisation during the 12-month study period vs those unvaccinated. Vaccine effectiveness tended to be lower during Omicron variant circulation.

**What are the implications of your findings for public health?**

Monitoring COVID-19 VE in population subgroups, such as children and adolescents, is important to inform future vaccination strategies. Our results showed that vaccination protected children against severe COVID-19 during Delta- and Omicron-circulating periods. We also highlighted valuable use of electronic health registeries to conduct VE studies in specific subgroups where severe outcomes were rare, vs more complex approaches using primary data.

## Introduction

More than 4 years after the declaration of the COVID-19 pandemic in March 2020, COVID-19 had considerable impact on the population health, with more than 770 million cases and 7 million deaths reported worldwide [1]. The majority of cases with severe COVID-19 were reported in the older adult population aged more than 65 years, and the risk of mortality in paediatric patients was estimated to be under 1% [2].

Between December 2020 and 2021, the European Commission authorised several COVID-19 vaccines for use in the paediatric population, including mRNA vaccines (Comirnaty, BNT162b2 mRNA, BioNTech-Pfizer; and Spikevax, mRNA-1273, Moderna) approved for use in children and adolecents aged 5–17 years, and protein-based vaccines (Nuvaxovid, NVX-CoV2373, Novavax) approved for use in children and adolecents aged 12–17 years [3]. In children aged 5–11 years, the vaccination scheme consisted of two low doses of mRNA vaccine [3]. Although COVID-19 was mostly a mild disease in children [2], many criteria were taken into account before the vaccine was recommended, including the low but non-negligible risk of severe outcomes in children. Other indirect benefits were considered, namely the reduction of disease transmission and minimising the interruption of academic activities of children or school reopening [4,5]. In most European countries, vaccination campaigns occurred during summer 2021 for children and adolecents aged 12–17 years and during late autumn 2021/early winter 2022 for children aged 5–11 years, in accordance with an age-decreasing strategy for vaccination roll-out.

Real-world studies on COVID-19 vaccine effectiveness (VE) against severe outcomes have been performed in various age groups, for different variants of concern (VOCs), different vaccination strategies (e.g. comparing primary vaccination and boosters) and at different times since vaccination [6-21]. These studies have shown that VE against hospitalisation varies between 49% and 74% [8,16] in children aged 5–11 years and is higher than 80% [8,10,15-18] in adolescents aged 12–17 years. Most paediatric studies evaluated VE up to 6 months after vaccination [6-8,20,21], with only a few studies examining up to 12 months post-vaccination [9,10,15,19]. Estimates were mostly available for mRNA vaccines, including Comirnaty [7-10] and Spikevax [15,18,19] but also for the inactivated vaccine CoronoVac (Sinovac Life Sciences) [6,21].

Few of the aforementioned real-world studies took place in the European Union/European Economic Area (EU/EEA) where local epidemiology may differ, with different response measures to COVID-19 and vaccine strategies that could have been adopted to target different age groups over different calendar periods (and, consequently, different VOCs). Thus, we aimed to estimate VE with two COVID-19 vaccine doses against hospital admission due to COVID-19 in children and adolescents aged 5–17 years eligible for vaccination, among those with and without documented SARS-CoV-2 previous infection. Among 5–17-year-olds with previously documented SARS-CoV-2 infection, VE for one vaccine dose was also estimated. Additionally, we aimed to estimate VE by time since vaccination, VOC and vaccine brand among those not previously infected.

## Methods

### Study design, setting, population and period

This study was developed within the VEBIS platform (Vaccine Effectiveness, Burden and Impact Studies), under the project ‘Vaccine effectiveness and the impact of COVID-19 vaccines through routinely-collected exposure and outcome data using health registries’ [22]. We implemented retrospective historical multicountry cohort studies of children and adolescents using data collected routinely by electronic health registries (EHR) in six study sites (Denmark, Norway, Italy, Luxembourg, Navarre (Spain) and Portugal). The study period was defined as 12 months, starting from the beginning of the vaccination campaign in each study site and age group. Supplementary Table S1 presents the vaccination calendar for age group and study site.

Detailed information on the study design and populations has been published elsewhere [23]. Briefly, we used a fixed cohort approach, defining the eligible population by age at the vaccination campaign's start. Age was categorised into two age groups: 5–11 years and 12–17 years. Eligible individuals were in EHRs at the beginning of the vaccination campaign, aged 5–17 years, residents of European countries or regions with participating study sites and eligible for COVID-19 vaccination. To estimate VE among individuals with previous SARS-CoV-2 infection, the study population included only individuals with a previously documented SARS-CoV-2 infection more than 90 days since the previous infection and eligible for COVID-19 vaccination. 

For variant-specific VE, the study period was restricted to the weeks when a particular SARS-CoV-2 VOC was predominant in each study site. Predominant periods were defined when at least 80% of the sequenced samples reported to the Global Initiative on Sharing All Influenza Data (GISAID) or to The European Surveillance System (TESSy) corresponded to the VOC of interest, which included Delta, Omicron BA.1/BA.2 and Omicron BA.4/BA.5 variants [24].

### Vaccination status, outcome and stratification

To determine the vaccination status, we analysed the number of COVID-19 vaccine doses administered to children and adolescents as a time-changing variable. We defined person-time of individuals as unvaccinated (no registered COVID-19 vaccine dose), vaccinated with one Comirnaty or Spikevax vaccine dose (only of interest in individuals with previous SARS-CoV-2 infection) and vaccinated with two Comirnaty or Spikevax vaccine doses. Children aged 5–11 years were vaccinated with the paediatric formulation [3], and adolescents aged 12–17 years with the adult formulation. Vaccine doses were administered over the study period following each country-specific recommendation and calendar time for each age group [22]. Person-time exposure to first and second doses between administration and completion status (i.e. 0–13 days after administration) was excluded from the analysis.

Time since completion of the vaccination was grouped as follows: 14–89 days, 90–179 days and 180–365 days. For brand-specific VE, we only included individuals who received two doses of the same paediatric Comirnaty (or Spikevax) vaccine formulation with recommended time between doses as per relevant country guidelines. In Supplementary Table S2, a description of eligibility criteria is presented, for both individuals with and without previous infection.

The outcome of interest was defined as admission to a hospital with a laboratory-confirmed SARS-CoV-2 infection using reverse-transcription PCR (RT-PCR), or antigen test, within 24 h post-admission or up to 14 days pre-admission. Clinical admission criteria include severe acute respiratory infection or coded as ‘COVID-19’. The outcome definition by study site is provided in Supplementary Table S3.

### Data sources and linkage, confounding variables and cohort construction

We used EHRs available at each study site. Four types of databases were linked using a deterministic approach to build the cohort of participants and their vaccine exposure history: (i) the reference population database (census database, health coverage database, etc.) with the individual records of the study target population (the sources of information on the reference population are provided in Supplementary Table S4); (ii) the vaccination registry database, including vaccination dates, doses and brands; (iii) the hospital admission database and COVID-19 laboratory-confirmed infection records; and (iv) databases used to retrieve information on confounding and stratification variables, i.e. primary healthcare records, medical prescriptions, hospital registries.

Potential confounding variables included sex, age (categorised into age groups 5–9, 10–11, 12–15 and 16–17 years) and socioeconomic indicators at the individual or regional level, region, country of birth and comorbidities, which were categorised into a three-level factor: no comorbidities, low-risk (non-immunocompromising condition) and high-risk comorbidities (any immunocompromising condition).

All children and adolescents were followed from the start of the vaccination campaign (or for variant-specific VE from the start of the VOC period) for each age group until: (i) outcome occurrence; (ii) the end of the study (12 months after the start of the campaign, or for variant-specific VE until the end of the VOC period); (iii) date of death; (iv) discontinuation in the administrative database; (v) SARS-CoV-2 laboratory diagnosis without hospitalisation occurrence; or (vi) receiving a booster vaccine dose.

### Analysis plan, vaccine effectiveness, study level adjustment for confounding and pooled analyses

The distribution of participants' baseline characteristics by vaccine status at the end of the follow-up period was described using absolute and relative frequencies. We estimated VE as one minus the confounder-adjusted hazard ratio (aHR) of COVID-19 hospitalisation between vaccinated with two doses (or one dose) vs unvaccinated. We used Cox proportional hazards regression models to estimate aHRs, considering the outcome as the time until the first hospitalisation because of COVID-19 and the exposure being the time-dependent vaccination status (one or two doses, time since vaccination and vaccine brand). To adjust for confounding, the variables age, sex, region, comorbidities and health-seeking behaviour were also included as covariates in the model. A full descrition of variables used for adjustment in each study site are presented in Supplementary Table S5. Given the expected low frequency of severe outcomes in each study site and the need to adjust for several potential confounders, an alternative approach applying an Inverted Probability of Treatment Weighting (IPTW) was used in Portugal, according to the methods described in the study protocol [23]. Additionally, study sites could participate in one or more objectives according to data availability and number of events. For example, only four study sites were included for the principal objective: Denmark, Italy, Navarre (Spain) and Portugal. Analyses performed are summarised in Supplementary Table S6.

Country-specific aHRs and standard errors for the effect of COVID-19 vaccination in the log scale obtained from the study sites were combined in a meta-analysis model. Considering the expected heterogeneity, we used a random-effects model for the main analysis and a fixed-effects model for sensitivity analysis.

## Results

We included 4,144,667 and 3,861,841 participants aged 5–11 years and 12–17 years, respectively. At the study site level, the sample size varied from 35,064 to 3,156,389 in the age group 5–11 years and from 17,703 to 2,802,810 in those 12–17 years. Considering both age groups, Luxembourg was the study site with the lowest sample size, and Italy with the highest. The proportion of males and females was similar in both age groups, with a slightly higher proportion of males (Table 1). Both age groups were skewed to the younger age in both children and adolescent age groups (children aged 5–9 years: 70.9% and adolescents aged 12–15: 63.5%). Supplementary Tables S7 and S8 present the sample distribution in each study site.

**Table:** Characteristics of the paediatric sample by age group at the end of the 12-month study period, VEBIS EHR network, six European countries, 2021–2022 (n = 8,006,508)

Characteristics		Age group			
		5–11 years		12–17 years	
		n	%	n	%
Total		4,144,667	100	3,861,841	100
**Sex **					
Male		2,129,318	51.4	1,993,004	51.6
Female		2,015,349	48.6	1,868,837	48.4
**Age group (years)**					
5–11 age group	5–9	2,936,952	70.9	NA	
	10–11	1,207,715	29.1		
12–17 age group	12–15	NA		2,452,487	63.5
	16–17			1,409,354	36.5
**Country of birth**					
Native		396,186	92.7	471,467	90.2
Non-native		30,994	7.3	51,030	9.8
Missing		0		15	
Not reported^a^		3,717,487		3,339,329	
**Nationality**					
National		343,754	88.9	352,783	92.5
Non-national		43,076	11.1	28,735	7.5
Missing		41		16	
Not reported^b^		3,757,796		3,480,307	
**Comorbidities **					
No comorbidity		845,550	88.7	364,001	88.5
Non-immunocompromising comorbidity		103,307	10.8	45,897	11.2
Immunocompromising comorbidity		4,357	0.5	1,207	0.3
Not reported^c^		3,191,453		3,450,736	

NA: not applicable; VEBIS EHR: Vaccine Effectiveness Burden and Impact Studies- Electronic Health Registries.

^a^ Italy and Portugal did not report data on country of birth.

^b^ Italy, Navarre (Spain), Norway and Portugal did not report data on nationality.

^c^ Italy and Luxembourg did not report any data on comorbidities.

**Figure 1:**
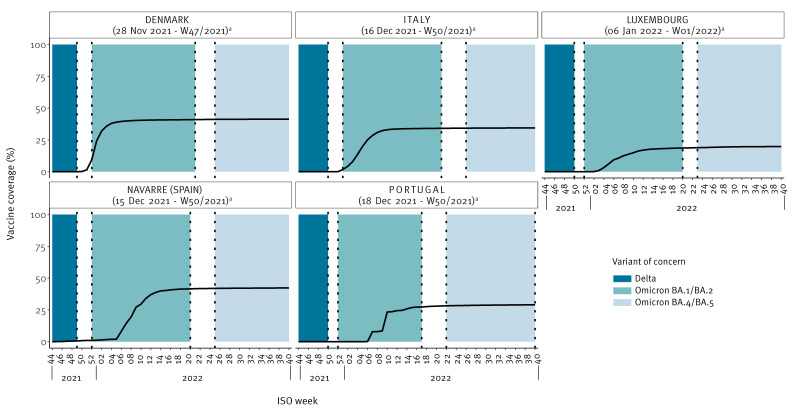
Weekly evolution of vaccine coverage for COVID-19 complete vaccination series for the 5–11-year age group within the VEBIS EHR network, six European countries, 2021–2022 (n = 4,144,667)

**Figure 2:**
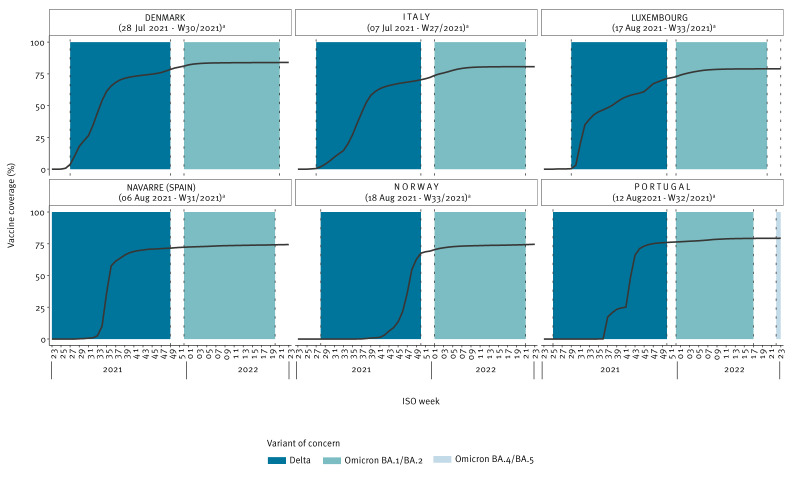
Weekly evolution of vaccine coverage for COVID-19 complete vaccination series for the 12–17-year age group within the VEBIS EHR network, six European countries, 2021–2022 (n = 3,861,841)

The coverage of two vaccine doses was consistently lower in children 5–11 years than in adolescents aged 12–17 years (Figure 1 and Figure 2). At the end of the follow-up period, coverage varied between 15.3% (Luxembourg) and 47.5% (Navarre (Spain)) in 5–11-year-olds, and between 59.7% (Luxembourg) and 84.9% (Portugal) in 12–17-year-olds. Figures 1 and 2 illustrate distinct periods of predominance for the VOCs under study. The Delta variant was prevalent at the outset of the vaccination campaign for adolescents aged 12–17 years. Conversely, the Omicron BA.4/BA.5 variant emerged predominantly towards the conclusion of the study period among children aged 5–11 years.

VEBIS EHR: Vaccine Effectiveness Burden and Impact Studies- Electronic Health Registries.

^a^ Date represents the start of the vaccination campaign.

Each study site is represented in a panel, displaying the date and ISO week of the start of the vaccination campaign.

VEBIS EHR: Vaccine Effectiveness Burden and Impact Studies- Electronic Health Registries.

^a^ Date represents the start of the vaccination campaign.

Each study site is represented in a panel, displaying the date and ISO week of the start of the vaccination campaign. Norway only reported data for adolescents between 16 and 17 years.

### Vaccine effectiveness

#### Individuals without previously documented infection (two vaccine doses)

**Figure 3:**
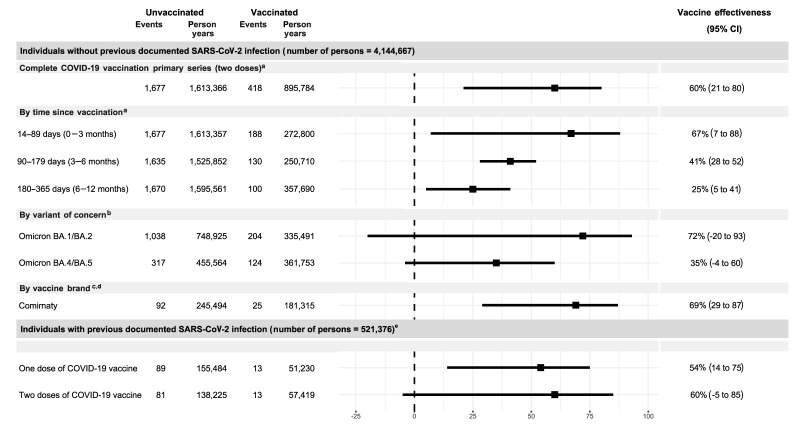
Pooled vaccine effectiveness with the number of events and person-years for children aged 5–11 years, within the VEBIS EHR network, four European countries, 2021–2022 (n = 4,144,667)

CI: confidence interval; SARS-CoV-2: severe acute respiratory syndrome coronavirus 2; VEBIS EHR: Vaccine Effectiveness Burden and Impact Studies- Electronic Health Registries.

^a^ Denmark, Italy, Navarre (Spain) and Portugal were included in the analyses.

^b^ Denmark, Italy and Portugal were included in the analyses.

^c^ Denmark, Navarre (Spain) and Portugal were included in the analyses.

^d^ No estimates for Spikevax were reported for the 5–11-years cohort.

^e^ Italy, Navarre (Spain) and Portugal were included in the analyses.

**Figure 4:**
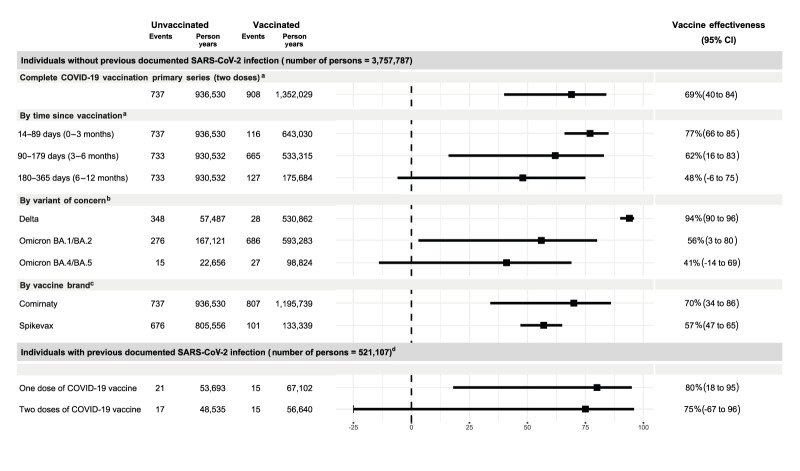
Pooled vaccine effectiveness with number of events and person-years for adolescents aged 12–17 years, within the VEBIS EHR network, four European countries, 2021–2022 (n = 3,861,841)

CI: confidence interval; SARS-CoV-2: severe acute respiratory syndrome coronavirus 2; VEBIS EHR: Vaccine Effectiveness Burden and Impact Studies- Electronic Health Registries.

^a^ Denmark, Italy, Navarre (Spain) and Portugal were included in the analyses.

^b^ Denmark, Italy and Portugal were included in the analyses.

^c^ Denmark, Navarre (Spain) and Portugal were included in the analyses.

^d^ Italy, Navarre (Spain) and Portugal were included in the analyses.

Figures 3 and 4 illustrate the VE estimates according to our objectives. We did not report VE estimates for Luxembourg because of the low number of hospitalisations. Similarly, Norway did not report data for ages 5–11 years, as vaccination was only recommended for children with underlying medical conditions. In the age group 12–17 years, Norway contributed data on adolescents aged 16–17 years, as different recommendations were issued for 12–15 and 16–17 year-olds. Thus, to minimise heterogeneity, pooled VE estimates do not incorporate data from Norway but were included in the sensitivity analysis. VE estimates per study site and pooled estimates with Norway are available in Supplementary Figures S1 and S4. 

Overall, in age groups 5–11 years and 12–17 years without previous infection, pooled VE estimates for two vaccine doses was 60% (95% CI: 21 to 80) and 69% (95% CI: 40 to 84), respectively. The median time since vaccination ranged from 49 to 292 days in the 5–11-year age group and from 140 to 293 days for the 12–17-year group. The study site specific descriptives of the time since vaccination and time since previous infection are provided in Supplementary Table S9. Vaccine effectiveness waned with time since vaccination in both age groups: from 67% to 25% in the 5–11-year age group and from 77% to 48% in the 12–17-year age group, 14–89 days and 180–365 days after vaccine administration respectively. Denmark, Italy, Navarre (Spain) and Portugal were included in the overall and waning analyses. 

In those 5–11 years, VE during the Omicron BA.1/BA.2 and BA.4/BA.5-dominant period was respectively 72% (95% CI: −20 to 93) and 35% (95% CI: −4 to 60) (Figure 3). No estimates for Delta were reported for the 5–11-year-old cohort, as they were not eligible for vaccination during the period of Delta circulation. In children aged 12–17 years (Figure 4) we observed higher VE during the Delta dominant circulating variant period (VE: 94%, 95% CI: 90 to 96) than Omicron BA.1/BA.2 (VE: 56%, 95% CI: 3 to 80) and BA.4/BA.5 (VE: 41%, 95% CI: −14 to 69). Denmark, Italy and Portugal were included in the VOC analyses. The VE for two doses of the Comirnaty vaccine was 69% (95% CI: 29 to 87) and 70% (95% CI: 34 to 86) in age groups 5–11 years and 12–17 years, respectively. Denmark, Navarre (Spain) and Portugal were included in the vaccine brand analyses. 

#### Individuals with previously documented infection

Overall, in age groups 5–11 years and 12–17 years with a previously documented SARS-CoV-2 infection, pooled VE estimates for one vaccine dose were respectively 54% (95% CI: 14 to 75) and 80% (95% CI: 18 to 95). We found more imprecise VE estimates for two vaccine doses, 60% (95% CI: −5 to 85) and 75% (95% CI: −67 to 96) in age groups 5–11 years and 12–17 years, respectively. Italy, Navarre (Spain) and Portugal were included in these analyses.

## Discussion

The low incidence of hospitalisations due to COVID-19 among children and adolescents aged 5–17 years warrants the implementation of studies with large sample sizes to estimate VE against COVID-19 hospitalisation. We found that two COVID-19 vaccine doses conferred moderate (up to 69%) long-term protection against hospitalisation in individuals of this age group without previous infection. On the other hand, one vaccine dose in the population with previously documented infection also had moderate to high (54 to 80%) long-term protection (up to 365 days). As expected, based on previous studies, we observed a decline in VE over time, with higher VE estimates observed in the first 3 months after immunisation. Considering the 12–17-year-old population, no significant VE differences were observed between the mRNA vaccines, Comirnaty and Spikevax, offered during the vaccination campaign. Finally, when comparing VE during different VOCs predominant circulation periods, for 12–17 years, we observed higher VE when Delta was the predominant variant in circulation compared with Omicron BA.1/BA.2 and BA.4/BA.5.

The comparison of VE estimates obtained in this study with other studies is limited, as most had shorter follow-ups after vaccination (e.g. up to 6 months), except for two studies in the US (with a follow-up of ca 8 [8] and another10 months [16]) and one multicountry study conducted in four Nordic European countries (follow-up up to 12 months [15]). Considering the studies with a longer follow-up period, VE against hospitalisations ranged from 40% (95% CI: 9–60) [16] to ca 83% (95% CI: 63.6–100) in adolescents [15]. Our results are within the range of published estimates, although not totally comparable, as we cover different periods of VOC circulation and include individuals without known previous infection.

For adolescents aged 12–17 years, VE among those with previous infection was 75–80%, indicating a vaccination benefit after a previous infection. The VE estimates were similar in individuals without previous infection who received two vaccine doses and those who received one vaccine dose, indicating the low added value of two doses in individuals with previous infection with SARS-CoV-2. This result could be important in designing future vaccination programmes, particularly in periods with limited availability of vaccines.

Another critical aspect for planning future vaccination campaigns is the potential waning of vaccine protection over time since vaccination. In a meta-analysis published in 2022, authors reported a VE decline 3 months post-immunisation [25]. Our long-term follow-up period of 12 months indicates that VE was highest in the first 3 months after post-immunisation (67–77%). Thereafter, VE waned to 41–62% after 3–6 months and 25–48% 6 or more months after vaccination. Although this decline was reported by other studies [8,21], others reported no significant waning over time [7,14-17]. Potential factors that could explain these results are the shorter follow-up periods precluding stratified analysis within the 3 months after vaccination [7,14,17], and the specific VOC in circulation at that time.

We also evaluated VE in different periods of Delta and Omicron (and respective sub-variants) circulation. In adolescents aged 12–17 years, we obtained higher VE estimates during Delta circulation (94%) than during Omicron BA1/2 (56%) or Omicron BA4/5 period (41%). High VE against hospitalisation during the Delta circulation period was reported in some studies [10,16], and lower VE (ranging from 40% to 78%) against hospitalisations during Omicron circulation [16,18]. Nevertheless, these VE estimates might be confounded by waning immunity. Additionally, the timing of vaccination implementation in different age groups and countries further challenges comparisons between age groups. Thus, future studies should aim to disentangle the potential waning effect from the lower VE against different VOCs as they are time-correlated. However, this might be challenging in studies with rare severe outcomes in children and adolescents.

Finally, we analysed the effectiveness of mRNA vaccine by product when a marketing authorisation was in place in the paediatric population. In the 12–17-year age group, we estimated VE of 70% for Comirnaty (similar estimate in 5–11 years) and 57% for Spikevax. Poukka et al. [15] obtained higher estimates for Spikevax vaccine (VE: 91%, 95% CI: 72.6 to 100) compared with Comirnaty vaccine (VE: 65.6%, 95% CI: 55.4 to 75.8) after 6 months of follow up. However, the study by Poukka et al. included more adolescents who completed their primary series with the Spikevax vaccine, allowing for better precision of the estimates. Spikevax was never the main product administered in any of the participating European study sites.

This study also has some limitations. Firstly, it is important to note that this study used EHR, thus the data collected were not intended for research purposes. This could lead to misclassification bias regarding vaccine status, outcomes and confounding variables. In some study sites, administrative datasets were only updated at the beginning of the seasonal campaign, which would not account for individuals who moved. Asymptomatic infections and self-tests performed at home might also not be recorded in the EHR, and individuals would be misclassified and included as individuals without previous SARS-CoV-2 infection, possibly underestimating VE. Vaccine protection may be over-estimated as individuals may have had hybrid protection from an undiagnosed/unreported infection and vaccination. Secondly, study sites had different testing policies and access to tests, which could have affected whether parents even considered testing their children (a description of testing policies in place during the study period is provided in Supplementary Table S10). Recommendations for vaccination also differed between countries. Although recommendations were similar for individuals without previous infection, these were asymmetrical between study sites considering a previous infection. Navarre (Spain) and Portugal, recommended one dose, except for children who were immunocompromised, while Denmark recommended two doses. Italy recommended one or two doses based on the time since the last infection. Nevertheless, pooled estimates for one dose VE among individuals with previous infection included only Italy, Navarre (Spain) and Portugal. However, time since the previous infection might also play a role, and this was not considered in the analysis (time since previous infection is described in Supplementary Table S9). There was also some heterogeneity considering the minimum recommended time interval between doses and the vaccination campaign start across study sites. Denmark started the vaccination campaign for adolescents 16–17 years old on 4 June 2021, while Norway started almost 11 weeks later on 18 August 2021. Different start dates might also represent different epidemiological scenarios, with differential natural immunity and risk perception across study site populations. Parents may have been more wary of vaccination against COVID-19 for their children and adolescents thus, vaccination might have been postponed in study sites with lower incidence rates [26]. These factors will affect mainly the vaccine coverage over time, so we do not expect that they will introduce bias in the VE estimates. Nevertheless, the fact that individuals receive the vaccine at different VOC predominance periods could affect the VE estimates if the effect of the vaccine differs by SARS-CoV-2 VOC. Each study site defined and adjusted for potential confounders [23]. We compared the crude and adjusted HR of the effect of vaccine doses in individuals without previous infection in each study site to assess the influence of the confounders included in the analysis (the COVID-19 hospitalisation hazard rate between vaccinated and unvaccinated without a previous SARS-CoV-2 infection, crude and fully adjusted for confounding is provided in Supplementary Tables S11 and S12). The variation between estimates was small and does not indicate the presence of confounding. It might be possible that there is residual confounding or that study sites could not properly identify and control for confounding. For instance, Italy and Portugal adjusted for the deprivation index, however, these variables were measured at the municipality level, which might be heterogeneous within some municipalities. This is also a limitation of EHR, as information is restricted, and information regarding confounders might not be possible to assess. One should be aware that estimates can vary in the presence of confounding.

Comparing VE estimates of different VOC periods can also be challenging since VOC predominance periods might be defined based on different thresholds during which other VOCs are circulating. Considering that individual data were unavailable, a threshold of 80% was the balance found between sample size and specificity in which we favoured specificity, leading to a more accurate VE but with less precision. Additionally, these VE estimates might be confounded by immunity waning and calendar time since the implementation of the vaccination campaign in different age groups and countries had some variations.

## Conclusion

This study highlighted that COVID-19 vaccines effectively prevented COVID-19 hospitalisation in over two thirds (69%) of 5–17-year-olds in European participating countries. This information is essential to discuss future recommendations in those age groups and research activities on the impact of the vaccination strategy. Despite the differences in systems and vaccination strategies among the countries, a multicountry approach was essential since the frequency of paediatric COVID-19 hospitalisations in the paediatric population is low. Monitoring COVID-19 VE in the paediatric/adolescent population using real-world data in evolving SARS-CoV-2 epidemiology is essential to inform public health policies. Real-world evaluations, as undertaken by this EHR network, provide estimates of VE against emerging variants, in populations not typically included in randomised controlled trials or against rare outcomes.

## Supplementary Data

2769000 Supplement
