# Supplementary material for: COVID-19 vaccine effectiveness in the paediatric population aged 5–17 years: a multicentre cohort study using electronic health registries in six European countries, 2021 to 2022
Source: Euro Surveill. 2025 Feb 27;30(8):2400450. doi: 10.2807/1560-7917.ES.2025.30.8.2400450 (PMC11869364; doi:10.2807/1560-7917.ES.2025.30.8.2400450)
Supplement: Supplement [file 24-00450_SOARES_Supplement.pdf]

This supplementary material is hosted by Eurosurveillance as supporting information alongside the article COVID-19 vaccine effectiveness in the paediatric population aged 5–17 years: a multicentre cohort study using electronic health registries in six European countries, 2021 to 2022 on behalf of the authors who remain responsible for the accuracy and appropriateness of the content. The same standards for ethics, copyright, attributions and permissions as for the article apply. Eurosurveillance is not responsible for the maintenance of any links or email addresses provided therein

Supplementary Table S1: Vaccination campaign rollout start date by study site and age groups.

Study site	16-17 yo	12-15 yo	5-11 yo
**Denmark**	**04/06/2021**	28/07/2021	28/11/2021
**Italy**	09/06/2021	**07/07/2021**	16/12/2021
**Luxembourg**	17/08/2021	**17/08/2021**	**06/01/2022**
**Navarre (Spain)**	02/08/2021	06/08/2021	**15/12/2021**
**Norway**	**18/08/2021**	*	*
**Portugal**	13/07/2021	12/08/2021	18/12/2021

*12-15 year olds only recommended one dose. Vaccination was not universally recommended among 5-11-year-olds in Norway. yo – years old.

Supplementary Table S2: Eligibility criteria for complete vaccination with and without previous infection during the study period.

Study site	Eligibility criteria for complete vaccination – two doses, without previous infection	Eligibility criteria for complete vaccination, with previous infection
**Denmark**	All children and adolescents. The interval between doses should have been no less than 19 days.	All children and adolescents received 2 doses.
**Italy**	All children and adolescents. The interval between doses should have been no less than 21 days between Comirnaty doses and 28 days between Spikevax doses.	Children and adolescents with a SARS-CoV-2 infection more than 12 months earlier were recommended 2 doses. Those with an infection 12 months or less earlier were recommended 1 dose only.
**Luxembourg**	All children and adolescents. The schedule was different depending on the health status of the children. The interval between doses should have been no less than 28 days.	All children and adolescents received 2 doses. The schedule was different depending on the health status of the children.
**Navarre (Spain)**	All children and adolescents. The interval between doses should have been no less than 19 days between Comirnaty doses and 22 days between Spikevax doses.	All children and adolescents received 1 dose.
**Norway**	In 2021, 2 doses were recommended for 16-17 years, 1 dose for 12-15 years, and 2 doses were recommended for all 5-17 years with underlying medical conditions. From the start of 2022, 2 doses were also available for 12-15 years. Recommendation for those with underlying risk, but (freely) available for all. The interval for 16-17-year-olds was recommended to be between 8-12 weeks between doses.	In 2021, 2 doses were recommended for 16-17 years, 1 dose for 12-15 years, and 2 doses were recommended for all 5-17 years with underlying medical conditions. From the start of 2022, 2 doses were also available for 12-15 years. Recommendation for those with underlying risk, but (freely) available for all.
**Portugal**	All children and adolescents. The interval between doses should have been no less than 19 days.	All children and adolescents received 1 dose. However, immunocompromised children and adolescents received 2 doses.

Supplementary Table S3: Outcome definition by site

Study site	Outcome definition
**Denmark**	Laboratory-confirmed infection 24 hours after hospital admission or 3 weeks before admission, lasting for a minimum of 24 hours and in which admission is classified with ICD-10 codes B342 and B972 or one of the sub-codes under these. The COVID-19-related ICD-10 codes have to be both primary diagnosis and action code.
**Italy**	Hospital admission with SARS-CoV-2 confirmed infection through RT-PCR or antigenic test and clinical manifestations of the respiratory tract or other organs directly associated with SARS-CoV-2 infection
**Luxembourg**	Hospitalised due to COVID-19 with a positive RT-PCR test within 14 days before and 24 hours after admission
**Navarre (Spain)**	Hospital admission with laboratory-confirmed COVID-19 infection by RT-PCR, reviewed by a medical doctor who concluded that the hospitalisation was due to COVID-19
**Norway**	Hospital admission due to COVID-19 with laboratory-confirmed SARS-CoV-2 infection
**Portugal**	COVID-19 is the main diagnosis in the discharge record

Supplementary Table S4: Source of information on the reference population

Study site	Source of information	Residency definition
**Denmark**	The Danish Civil Registration System (CPR)	Residency is defined as an individual who is registered as currently living in the country (as the main country of residency). It is not feasible to reside in Denmark for an extended duration without being officially registered as a resident.
**Italy**	Official population statistics provided by the Italian Institute of Statistics (Istat)	The database includes the size of the population residing in Italy at the beginning of each calendar year (1st of January) by sex, age and municipality/region. According to the Istat definition, the resident population is made up of people, of Italian and foreign citizenship, having habitual residence in the national territory even if temporarily absent.
**Luxembourg**	Administrative dataset collected by the national social security, IGSS ”Inspection génerale de la sécurité sociale”	The identification of non-residents is possible.
**Navarre (Spain)**	Administrative database	Residents covered by the Navarre Health Service. This Service covers 98% of the population in the region, with an unbiased distribution by sex and geographical areas.<br>The database contains variables that allow the identification of non-residents or temporary residents.
**Norway**	The National Population Register	Individuals must possess a valid national identity number and be registered in the National Population Registry as residents of Norway.
**Portugal**	National Health Service User (NHSU) dataset	Residents in mainland Portugal who had contact with the healthcare system in the previous three years.

Supplementary Table S5: Factors used to adjust for confounding in each study site and their operationalisation

Study site	Factors and covariates used to adjust for confounding	Variable operacionalisation
**Denmark**	Sex, age, region, and comorbidities	Comorbidities defined as no comorbidities, low-medium risk comorbidities (including the presence of at least one of the following conditions: Tuberculosis, Hematological, Coagulation, Diabetes, Adipose, Endocrinological, Ischemic heart disease, Heart problems, Chronic lung diseases, Alcoholic liver, Liver, Neurological, Kidney, Congenital, COPD, Contact regarding influenza vaccination, Cancer, Missing lung, Missing kidney, Alcohol), and high risk comorbidities (including the presence of at least one of the following conditions: HIV, Immunological, Irradiation, Transplantation)
**Italy**	Sex, age, region and deprivation index	Deprivation Index at the municipality level was provided by the Italian Institute of Statistics (Istat)
**Luxembourg**	Sex, age, country of birth, and nationality	
**Navarre (Spain)**	Sex, age, comorbidities/immunocompromised, and country of birth	Comorbidities defined as no comorbidities related to increased risk of COVID-19, low-medium risk comorbidities (including other comorbidities associated with the risk of COVID-19 but different from immunocompromising conditions: Diabetes, Severe obesity, Cancer, Stroke, Dementia, Kidney disease, Haematological cancers, Heart disease, Chronic respiratory disease, Liver disease and Rheumatic arthritis), and high risk comorbidities (Immunocompromising conditions)
**Norway**	Sex, age, living condition, country of residence, and comorbidities	Living condition correspond to individuals living in crowded conditions if the number of rooms is lower than the number of residents or one resident lives in one room, and the number of square metres (P-area) is below 25 sq. m. per person. If the number of rooms or the P-area is not specified, a household will be regarded as crowded if one of these criteria is met. https://www.ssb.no/a/metadata/conceptvariable/vardok/3462/en<br><br>Comorbidities defined as no comorbidities related to increased risk of COVID-19, low-medium risk comorbidities (including the presence of at least one of the following conditions: Chronic liver disease or significant hepatic impairment, Diseases requiring immunosuppressive therapy, Diabetes, Chronic lung disease including cystic fibrosis and severe asthma, which have required the use of high dose inhaled or oral steroids within the past year, Obesity with a body mass index (BMI) of ≥35 kg/m2, Dementia; Chronic heart and vascular disease (except for high blood pressure), and stroke), and high risk comorbidities (including the presence of at least one of the following conditions: Organ transplant, Immunodeficiency, Haematological cancer in the last five years, Other active cancers, Neurological or neuromuscular diseases that cause impaired cough or lung function (e.g., ALS and cerebral palsy), Chronic kidney disease, or significant renal impairment)
**Portugal**	Sex, age, region, European deprivation index, comorbidities, and number of SARS-CoV-2 tests performed in the previous year	European Deprivation Index at the municipality-level http://dx.doi.org/10.20344/amp.7387<br><br>Comorbidities defined as no comorbidities, low-medium risk comorbidities (including the presence of at least one of the following conditions without immunosuppression: anaemia, dementia, diabetes, cardiac disease, neuromuscular disease, rheumatologic disease, obesity, tuberculosis, stroke, pulmonary disease, asthma, liver disease and hypertension), and high risk comorbidities (including the presence of at least one of the following immunocompromising conditions: HIV, renal disease, and cancer)

Supplementary Table S6: Overview of the analyses developed

**Objective**	**Population**	**Exposed**	**Unexposed**
Estimate COVID-19 VE against hospital admission due to COVID-19	Children and adolescents (5-11 and 12-17 years old) without a previously documented SARS-CoV-2 infection	Vaccinated with two doses of the COVID-19 vaccine	Unvaccinated children and adolescents eligible for the COVID-19 vaccine
Estimate COVID-19 VE by time since completion of primary vaccination series – (0–89), (90–179), (180-365) days		Vaccinated with two doses of the COVID-19 vaccine, for the different time period	Unvaccinated children and adolescents eligible for the COVID-19 vaccine in each time period
Estimate COVID-19 VE by vaccine product – Cominarty and Spikevax		Vaccinated with two doses of the COVID-19 vaccine, for each vaccine product	Unvaccinated children and adolescents eligible for the COVID-19 vaccine for each vaccine product
Estimate COVID VE during the Delta predominance period		Vaccinated with two doses of the COVID-19 vaccine, during the Delta predominance period	Unvaccinated children and adolescents eligible for the COVID-19 vaccine during the Delta predominance period
Estimate COVID VE during the Omicron BA.1/BA.2 predominance period		Vaccinated with two doses of the COVID-19 vaccine, during the Omicron BA.1/BA.2 predominance period	Unvaccinated children and adolescents eligible for the COVID-19 vaccine during the Omicron BA.1/BA.2 predominance period
Estimate COVID VE during the Omicron BA.4/BA.5 predominance period		Vaccinated with two doses of the COVID-19 vaccine, during the Omicron BA.4/BA.5 predominance period	Unvaccinated children and adolescents eligible for the COVID-19 vaccine during the Omicron BA.4/BA.5 predominance period
Estimate COVID-19 VE against hospital admission due to COVID-19	Children and adolescents (5-11 and 12-17 years old) with a previously documented SARS-CoV-2 infection	Vaccinated with one dose of the COVID-19 vaccine	Unvaccinated children and adolescents eligible for the COVID-19 vaccine

Supplementary Table S7: Number of children included in the study by sociodemographic and clinical characteristics for the 5-11 age group, by study site within the VEBIS EHR network

	Denmark[1]	Italy[2]	Luxembourg[3]	Navarre (Spain)[4]	Portugal[5]
	N (%)	*N (%)*	*N (%)*	*N (%)*	*N (%)*
**Site**					
**Total**	351,807	3,156,389	35,064	40,309	561,098
**Sex**					
**Male**	180,825	1,622,345	17,839	20,689	287,620
	(51·4%)	(51·4%)	(50·9%)	(51·3%)	(51·3%)
**Female**	170,982	1,534,044	17,225	19,620	273,478
	(48·6%)	(48·6%)	(49·1%)	(48·7%)	(48·7%)
**Age group (years-old)**					
**5 to 9**	252,011	2,239,606	25,901	28,574	390,860
	(71·6%)	(71·0%)	(73·9%)	(70·9%)	(69·7%)
**10 to 11**	99,796	916,783	9,163	11,735	170,238
	(28·4%)	(29·0%)	(26·1%)	(29·1%)	(30·3%)
**Country of birth**					
**Native**	333,335	NR	26,211	36,640	NR
	(94·7%)		(74·8%)	(90·9%)	
**Non-native**	18,472	NR	8,853	3,669	NR
	(5·3%)		(252%)	(9·1%)	
**Nationality**					
**National**	325,100	NR	18,654	NR	NR
	(92·4%)		(53·2%)		
**Non-national**	26,707	NR	16,369	NR	NR
	(7·6%)		(46·7%)		
**Missing**	0	NR	41	NR	NR
			(0·1%)		
**Comorbidities**					
**No comorbidity**	309,803	NR	NR	33,803	501,944
	(88·1%)			(83·9%)	(89·5%)
**Low-medium risk comorbidities /non-immunocompromising**	41,062	NR	NR	6,352	55,893
	(11·7%)			(15·8%)	(10·0%)
**High-risk comorbidities/ immunocompromising**	942	NR	NR	154	3,261
	(0·3%)			(0·4%)	(0·6%)

Study started on: 1 – Nov 28th 2021, 2 – Dec 16th 2021, 3 – Jan 1st 2022, 4 – Dec 12th 2021, 5 – Dec 18th 2021; NR – not reported (unavailable data).

Supplementary Table S8: Number of adolescents included in the study by sociodemographic and clinical characteristics for the 12-17 age group, by study site within the VEBIS EHR network

	Denmark[1]	Italy[2]	Luxembourg[3+]	Navarre (Spain)[4]	Norway[5]*	Portugal[6]
	N (%)	*N (%)*	*N (%)*	*N (%)*	*N (%)*	*N (%)*
**Site**						
**Total**	363,831	2,802,810	17,703	36,924	104,054	536,519
**Sex**						
**Male**	186,870	1,449,967	9,305	19,089	53,828	273,945
	(51·4%)	(51·7%)	(52·6%)	(51·7%)	(51·7%)	(51·1%)
**Female**	176,961	1,352,843	8,398	17,835	50,226	262,574
	(48·6%)	(48·3%)	(47·4%)	(48·3%)	(48·3%)	(48·9%)
**Age group (years-old)**						
**12 to 15**	239,876	1,814,152	12,645	25,498	0	360,316
	(65·9%)	(64·7%)	(71·4%)	(69·1%)		(67·2%)
**16 to 17**	123,955	988,658	5,058	11,426	104,054	176,203
	(34·1%)	(35·3%)	(28·6%)	(30·9%)	(100·0%)	(32·8%)
**Country of birth**						
**Native**	338,108	NR	11,509	33,041	88,809	NR
	(92·9%)		(65·0%)	(89·5%)	(85·3%)	
**Non-native**	25,723	NR	6,194	3,883	15,230	NR
	(7·1%)		(35·0%)	(10·5%)	(14·6%)	
**Missing**	0	NR	0	0	15	NR
					(0·0%)	
**Nationality**						
**National**	342,751	NR	10,032	NR	NR	NR
	(94·2%)		(56·7%)			
**Non-national**	21,080	NR	7,655	NR	NR	NR
	(5·8%)		(43·2%)			
**Missing**	0	NR	16	NR	NR	NR
			(0·1%)			
**Comorbidities**						
**No comorbidity**	333,794	NR	NR	30,207	93,704	446,445
	(91·7%)			(81·8%)	(90·1%)	(83·2%)
**Low-medium risk comorbidities /non-immunocompromising**	29,546	NR	NR	6,552	9,799	85,893
	(8·1%)			(17·7%)	(9·4%)	(16·0%)
**High-risk comorbidities/ immunocompromising**	491	NR	NR	165	551	4,181
	(0·1%)			(0·4%)	(0·5%)	(0·8%)

Vaccination campaign for 16-17 years started on: 1 – Jun 4th 2021, 2 – Jun 9th 2021, 3 – Aug 17th 2021, 4 – Aug 2nd 2021, 5 – Aug 18th 2021, 6 – Jul 13th 2021. +data refers to primary series excluding participants who received a booster dose; *only reported data for adolescents between 16 and 17 years; NR – not reported (unavailable data)

Supplementary Table S9: Descriptives of the time since vaccination and time since previous infection for each age group, by study site within the VEBIS EHR network

**Time since vaccination**				
	**5-11**		**12-17**	
	**Mean (range)**	**Median (Q1-Q3)**	**Mean (range)**	**Median (Q1-Q3)**
**DN**	156 (0-331)	49 (15-309)	173 (0-330)	154 (133-196)
**IT**	241 (0-330)	292 (202-306)	147 (0-330)	140 (202-306)
**NV**	344 (16-360)	289 (274-306)	320 (0-320)	293 (287-298)
**PT**	235 (0-331)	281 (225-281)	212 (0-330)	211 (129-304)
**Time since previous infection**				
	**5-11**		**12-17**	
	**Mean (range)**	**Median (Q1-Q3)**	**Mean (range)**	**Median (Q1-Q3)**
**IT**	300 (91-662)	296 (243-377)	187 (91-496)	190 (131-232)
**NV**	313 (90-646)	328 (208-421)	244 (90-511)	273 (181-298)
**PT**	300 (90-713)	331 (179-376)	224 (90-552)	213 (189-257)

Time since vaccination corresponds to the 14 days after administering the second dose until censorship or event. Time since previous infection corresponds to the time since the date of the (previous) infection and the study start, i.e., the start of the vaccination campaign.

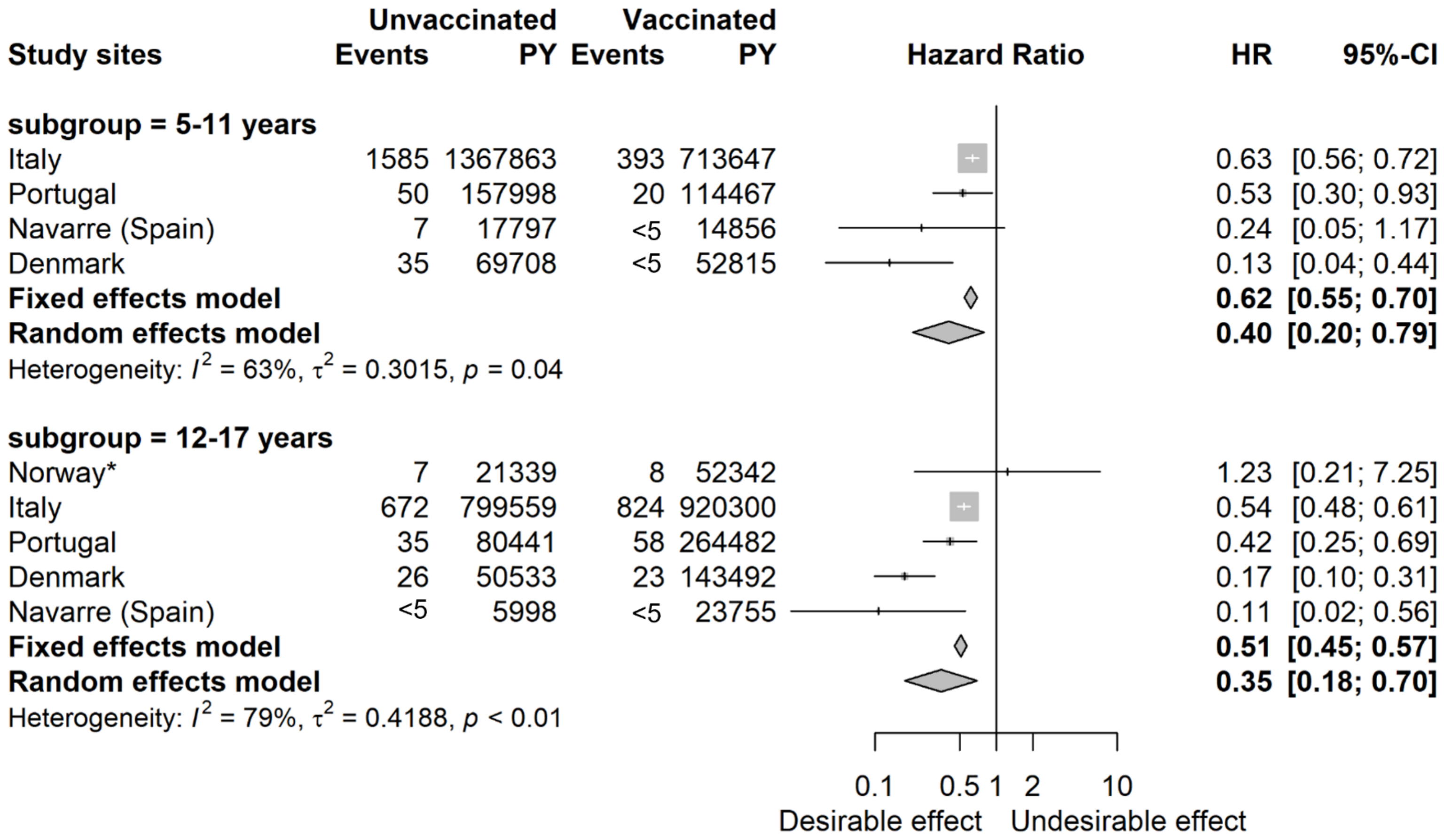

Supplementary Figure S1: Forest plot with study site-specific number of COVID-19 hospitalisations, person-years by vaccine status, confounder-adjusted hazard ratio of COVID-19 hospitalisation (and 95% confidence interval) between participants without previous SARS-CoV-2 infection vaccinated versus unvaccinated, and pooled estimates using random and fixed effects approach (with 95% confidence interval). (τ^2 heterogeneity between study sites estimates, I^2 proportion of the heterogeneity not explained by random variation)

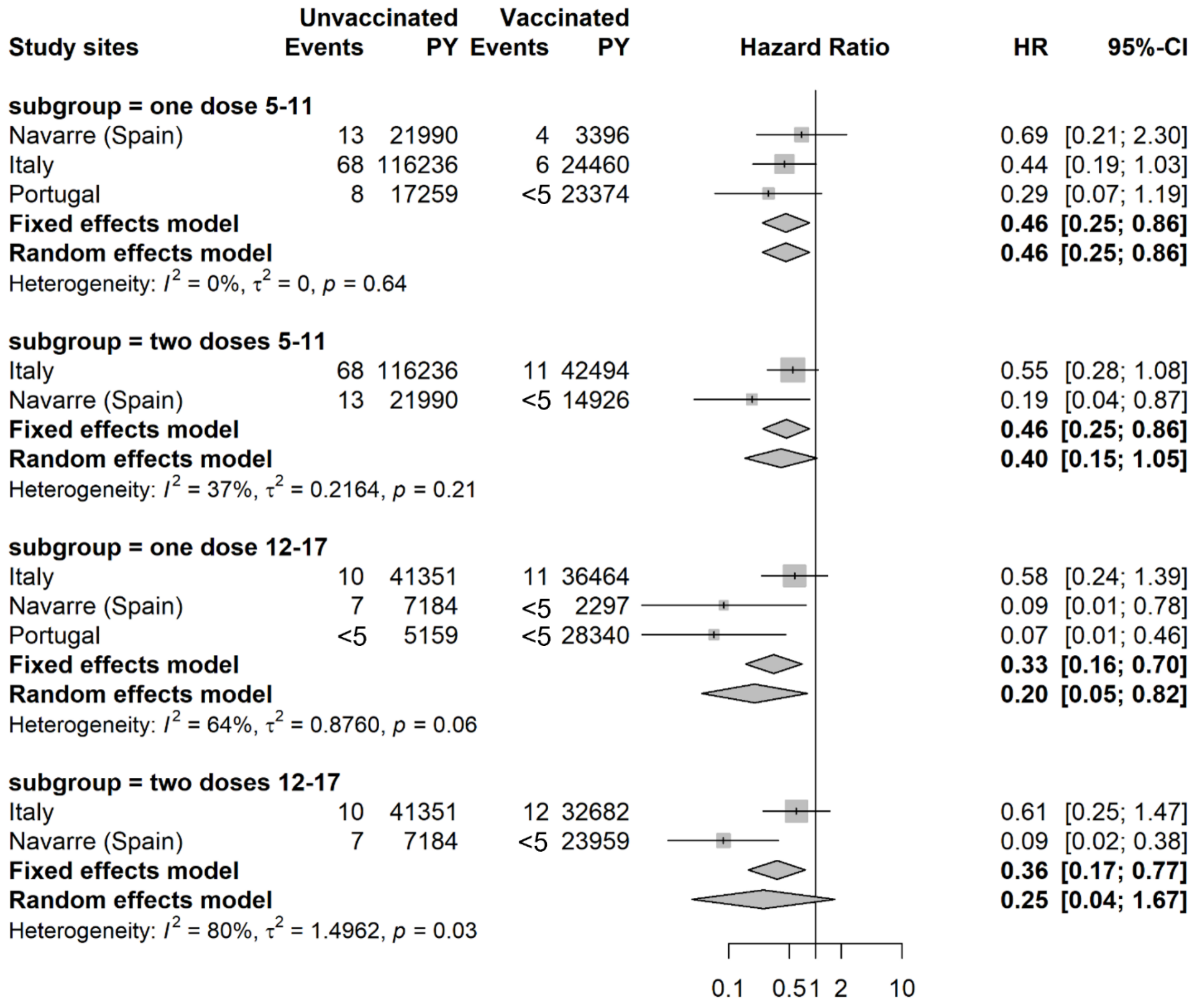

Supplementary Figure S2: Forest plot with study site-specific number of COVID-19 hospitalisations, person-years by vaccine status, confounder-adjusted hazard ratio of COVID-19 hospitalisation (and 95% confidence interval) between participants with previous SARS-CoV-2 infection vaccinated with one and two doses versus unvaccinated, and pooled estimates using random and fixed effects approach (with 95% confidence interval). (τ^2 heterogeneity between study sites estimates, I^2 proportion of the heterogeneity not explained by random variation)

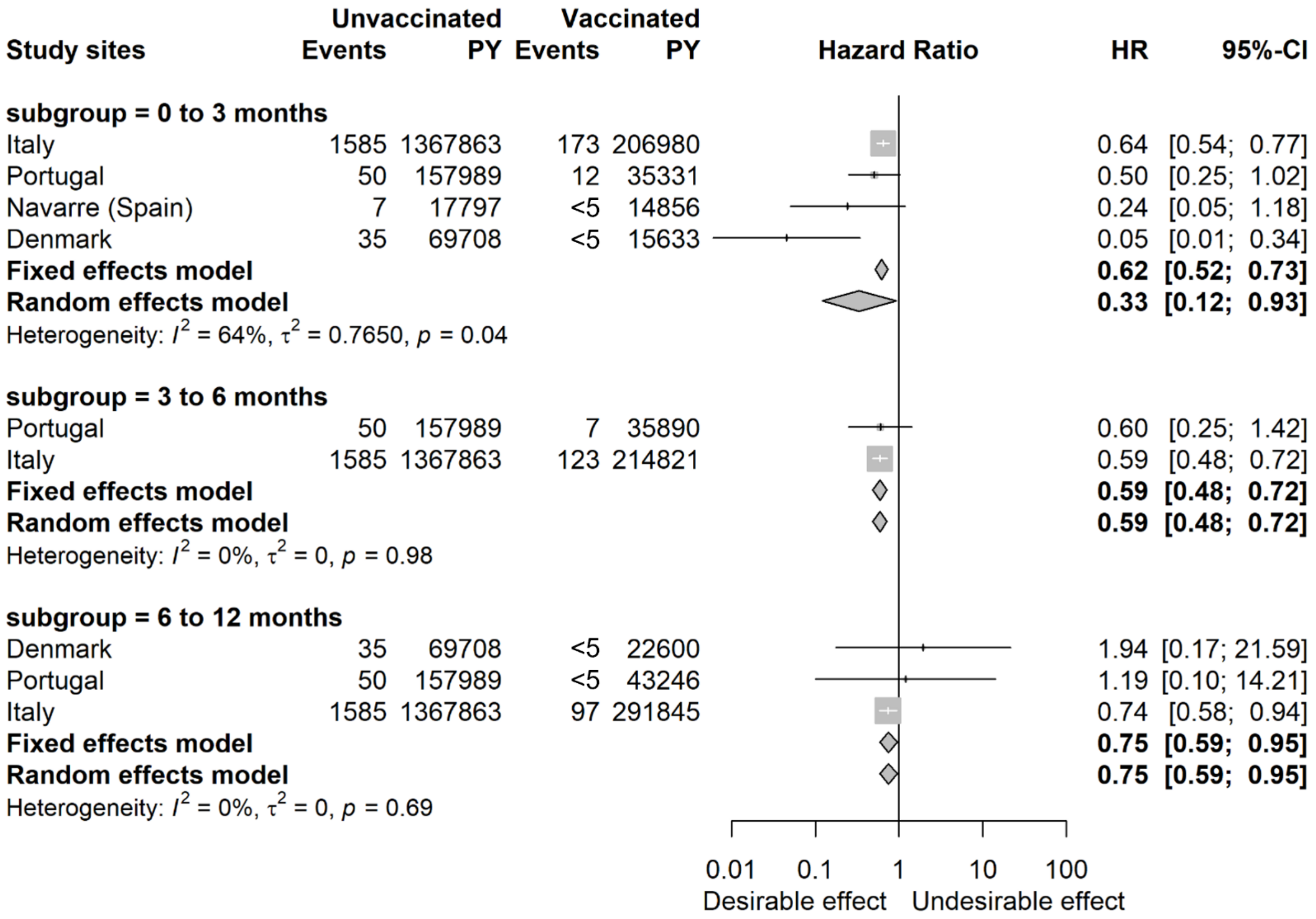

Supplementary Figure S3: Forest plot with study site-specific number of COVID-19 hospitalisations, person-years by vaccine status, confounder-adjusted hazard ratio of COVID-19 hospitalisation (and 95% confidence interval) between participants without previous SARS-CoV-2 infection vaccinated versus unvaccinated by time since vaccination, and pooled estimates using random and fixed effects approach (with 95% confidence interval), for the 5-11 cohort. (τ^2 heterogeneity between study sites estimates, I^2 proportion of the heterogeneity not explained by random variation)

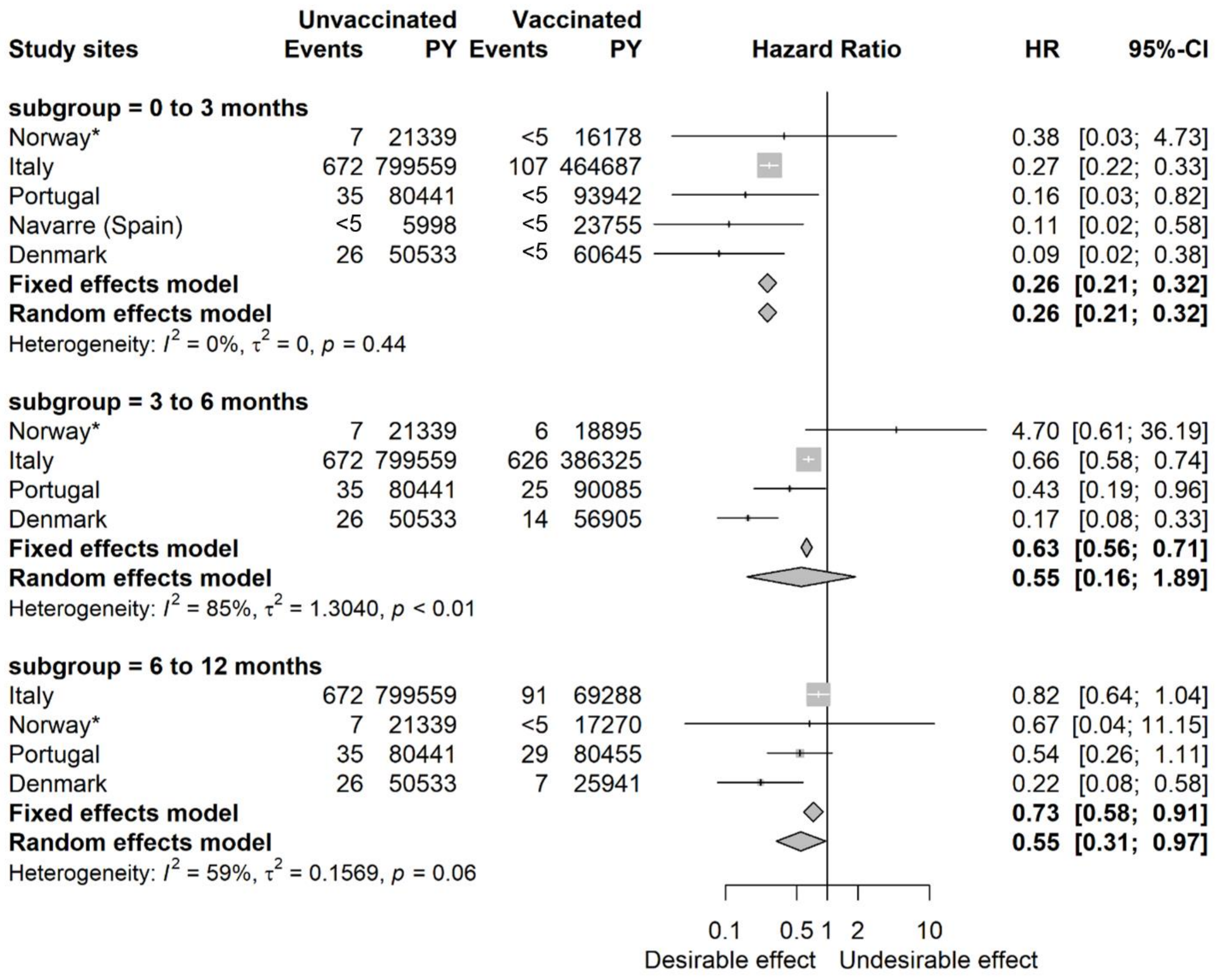

Supplementary Figure S4: Forest plot with study site-specific number of COVID-19 hospitalisations, person-years by vaccine status, confounder-adjusted hazard ratio of COVID-19 hospitalisation (and 95% confidence interval) between participants without previous SARS-CoV-2 infection vaccinated versus unvaccinated by time since vaccination, and pooled estimates using random and fixed effects approach (with 95% confidence interval), for the 12-17 cohort. (τ^2 heterogeneity between study sites estimates, I^2 proportion of the heterogeneity not explained by random variation)

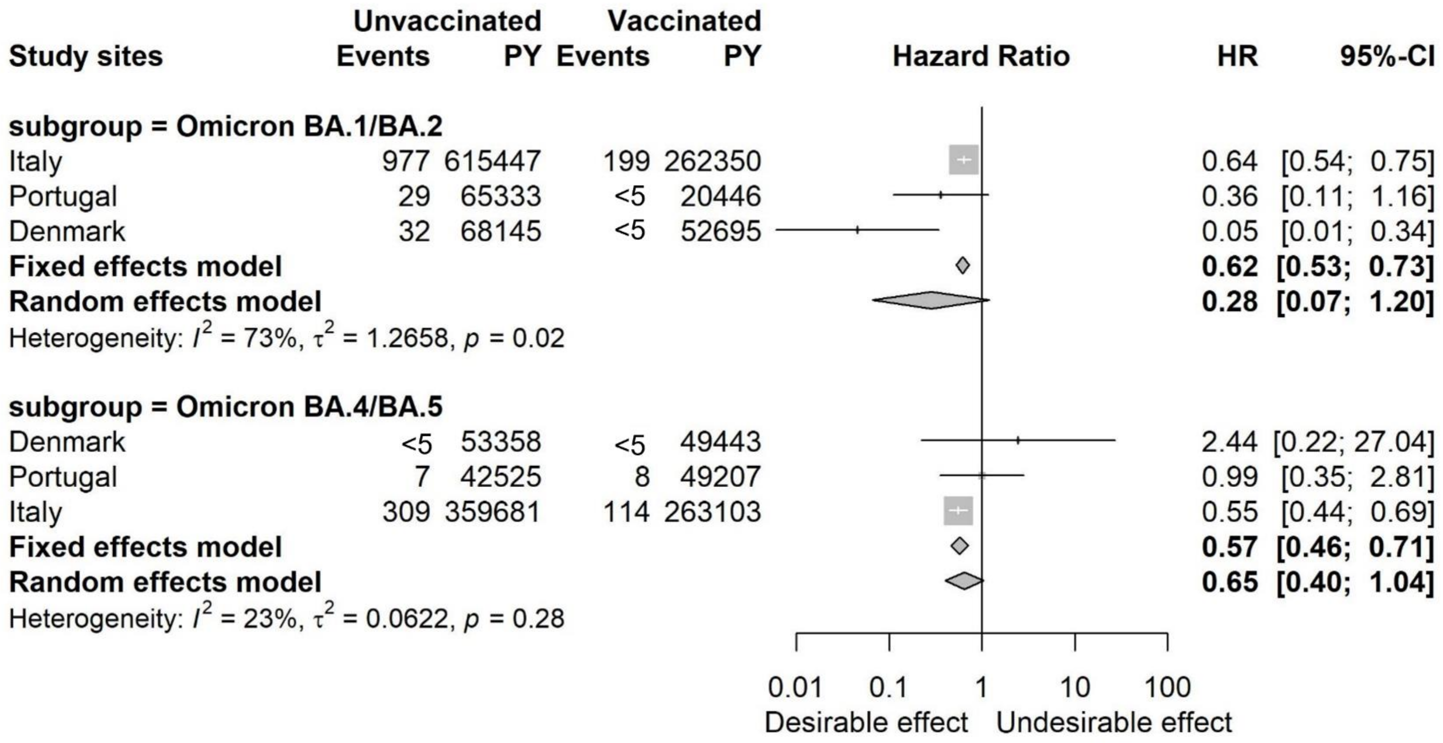

Supplementary Figure S5: Forest plot with study site-specific number of COVID-19 hospitalisations, person-years by vaccine status, confounder-adjusted hazard ratio of COVID-19 hospitalisation (and 95% confidence interval) between participants without previous SARS-CoV-2 infection vaccinated versus unvaccinated by variant of concern predominance period, and pooled estimates using random and fixed effects approach (with 95% confidence interval), for the 5-11 cohort. (τ^2 heterogeneity between study sites estimates, I^2 proportion of the heterogeneity not explained by random variation)

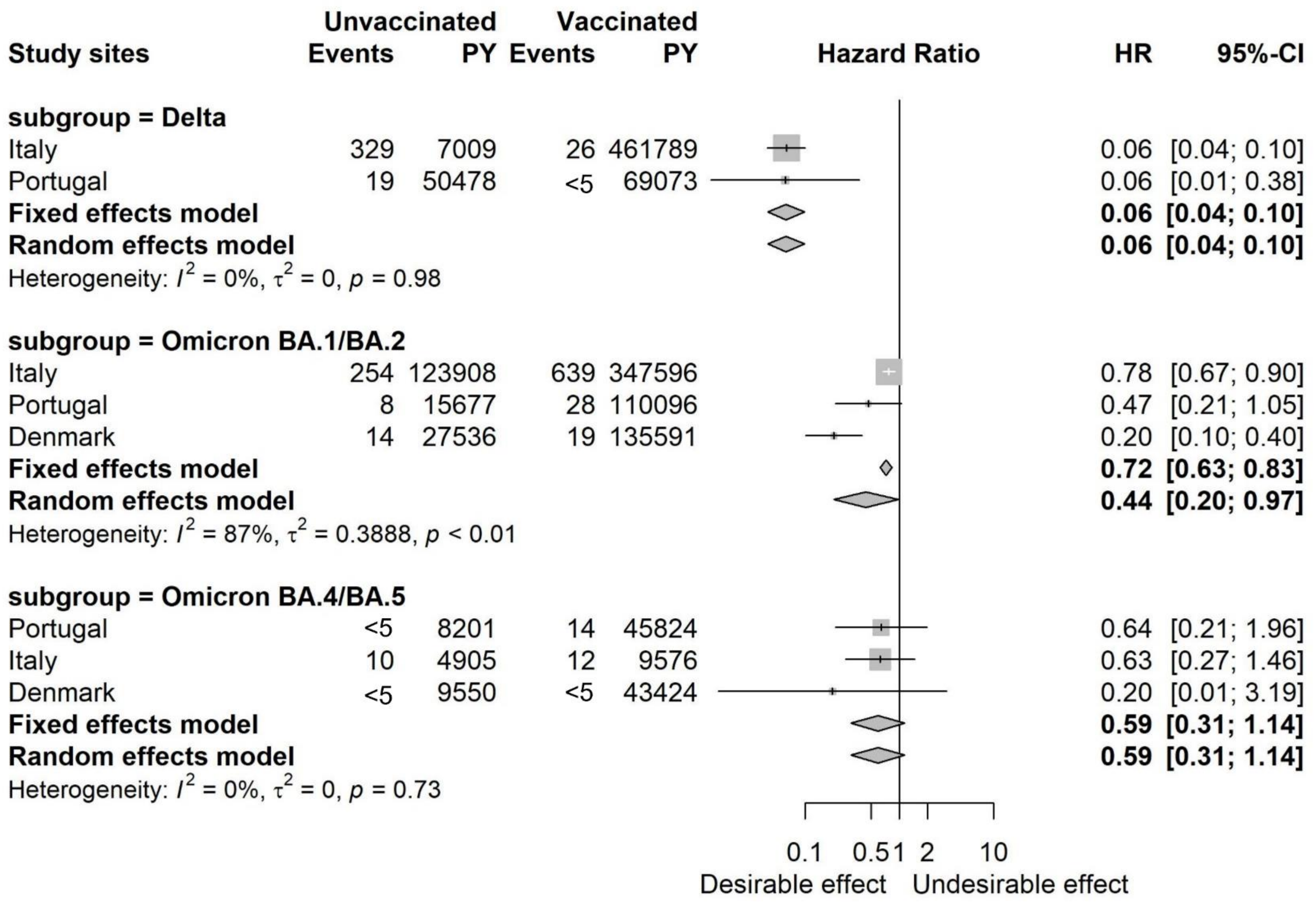

Supplementary Figure S6: Forest plot with study site-specific number of COVID-19 hospitalisations, person-years by vaccine status, confounder-adjusted hazard ratio of COVID-19 hospitalisation (and 95% confidence interval) between participants without previous SARS-CoV-2 infection vaccinated versus unvaccinated by variant of concern predominance period, and pooled estimates using random and fixed effects approach (with 95% confidence interval), for the 12-17 cohort. (τ^2 heterogeneity between study sites estimates, I^2 proportion of the heterogeneity not explained by random variation)

Study sites	Unvaccinated Events	Unvaccinated PY	Vaccinated Events	Vaccinated PY	HR	95%-CI
**subgroup = Comirnaty 5-11**						
Portugal	50	157989	20	114463	0.53	[0.30; 0.93]
Navarre (Spain)	7	17797	<5	14770	0.24	[0.05; 1.17]
Denmark	35	69708	<5	52083	0.13	[0.04; 0.44]
**Fixed effects model**					**0.39**	**[0.24; 0.64]**
**Random effects model**					**0.31**	**[0.13; 0.71]**
Heterogeneity: $I^2 = 56\%$, $\tau^2 = 0.2688$, $p = 0.10$						
**subgroup = Comirnaty 12-17**						
Norway*	7	21187	8	50497	1.27	[0.22; 7.39]
Italy	672	799559	724	787475	0.56	[0.50; 0.63]
Portugal	35	80441	58	263697	0.44	[0.27; 0.72]
Denmark	26	50533	23	143087	0.16	[0.09; 0.29]
Navarre (Spain)	<5	5998	<5	1479	0.07	[0.01; 0.56]
**Fixed effects model**					**0.53**	**[0.47; 0.59]**
**Random effects model**					**0.35**	**[0.16; 0.77]**
Heterogeneity: $I^2 = 82\%$, $\tau^2 = 0.5763$, $p < 0.01$						
**subgroup = Spikevax 12-17**						
Italy	672	799559	100	132825	0.43	[0.35; 0.53]
Navarre (Spain)	<5	5998	<5	515	0.28	[0.03; 2.70]
**Fixed effects model**					**0.43**	**[0.35; 0.53]**
**Random effects model**					**0.43**	**[0.35; 0.53]**
Heterogeneity: $I^2 = 0\%$, $\tau^2 = 0$, $p = 0.72$						

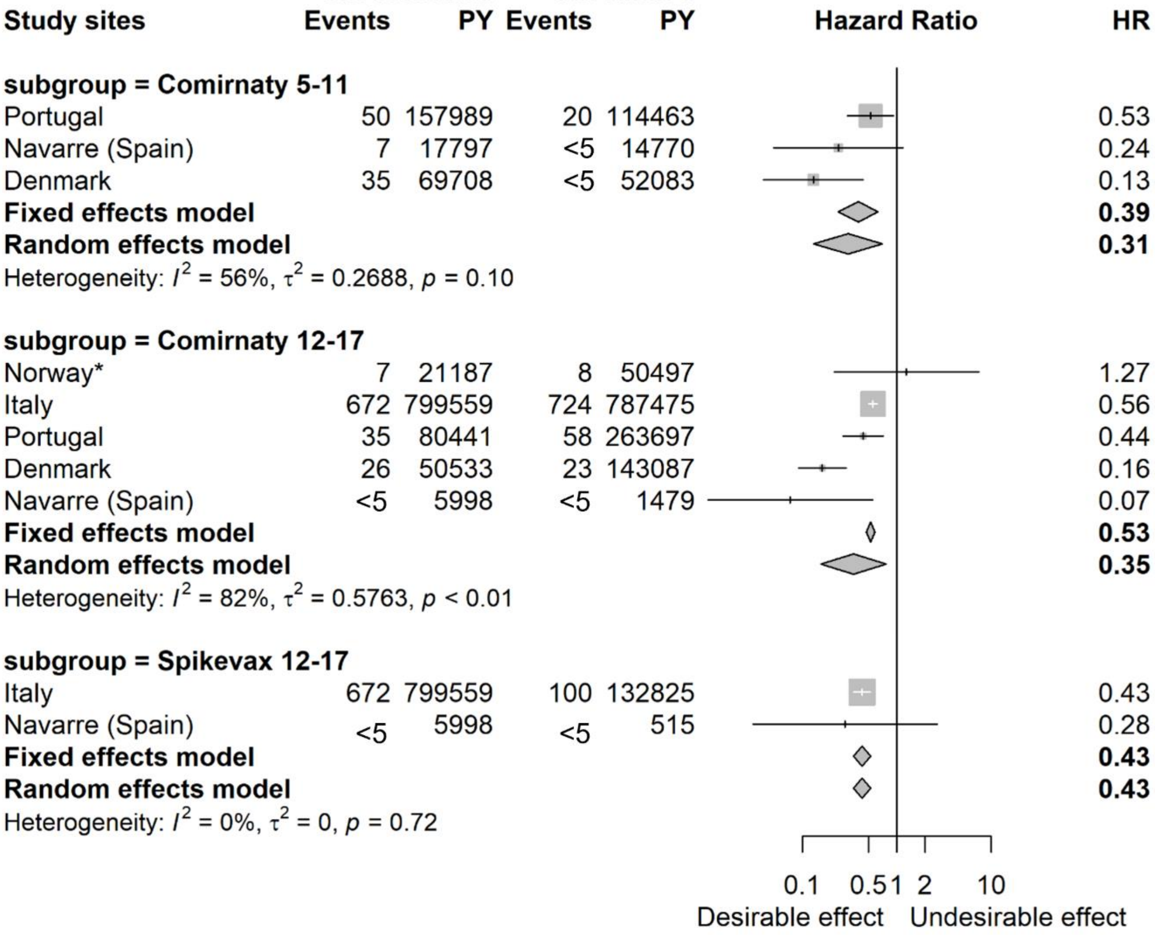

Supplementary Figure S7: Forest plot with study site-specific number of COVID-19 hospitalisations, person-years by vaccine status, confounder-adjusted hazard ratio of COVID-19 hospitalisation (and 95% confidence interval) between participants without previous SARS-CoV-2 infection vaccinated versus unvaccinated by vaccine brand, and pooled estimates using random and fixed effects approach (with 95% confidence interval). (τ^2 heterogeneity between study sites estimates, I^2 proportion of the heterogeneity not explained by random variation)

Supplementary Table S10: Testing policies in place during the study period in each study site

Study sites	Testing policies
**Denmark**	**Testing eligibility:** Children of all ages during the study period were eligible to be tested by PCR free of charge throughout the study period. However, accessibility to, and the use of, PCR testing facilities reduced significantly after March 2022, when the capacity of testing facilities was scaled down.<br>**Rapid antigen tests:** Although free-of-charge, readily accessible rapid antigen testing was widely utilised in Denmark alongside PCR testing among adults and adolescents during the study period. Rapid antigen tests were generally not recommended for use with children under the age of 12. The majority of tests conducted on children under 12 years of age were by PCR.<br>**Hospital COVID-19 screening practices:** Variations in hospital COVID-19 screening practices were of little concern due to the high sensitivity (hospital patients were routinely tested by PCR upon admission, especially if COVID-19 was suspected) and specificity of the outcome definition.<br>**Restrictions associated with COVID-19 vaccination**: From November 2021 to January 2022, many restrictions were in place to limit the spread of SARS-CoV-2 in schools, workplaces, public transport, public spaces, shops and restaurants, including the closure of schools (online learning only) for parts of December 2021. For limited periods during November 2021 and January 2022, some colleges and educational institutions required that +15-year-olds produce a so-called “corona-pas”, i.e. one of the following: (i) a recent negative test, (ii) a positive test result dated 14 to 180 days earlier, (iii) proof of having completed the primary covid-19 vaccination schedule.
**Italy**	**Testing eligibility:** Between December 2021 and October 2022, there were no official changes in the testing policy.<br>**Rapid antigen tests:** Rapid antigen tests were authorised before the start of the study period in January 2021. The absolute number of tests did not decrease during the Omicron wave (neither PCR nor rapid antigen tests). However, the relative percentage of PCR and rapid antigen tests decreased as there was a substantial increase in the number of self-tests. Tests were free if you had a prescription from your doctor. If you went to a pharmacy to have one done without a prescription, the cost was 8€ for those under 18 years old. This was fixed price from August 2021 until March 2022. Since January 2022, most cases have been diagnosed through self-administered rapid antigen tests.<br>**Hospital COVID-19 screening practices:** Between December 2021 and October 2022, there were also no changes in the official recommendations. All hospitals were strongly recommended to screen all patients on admission (or ED attendance).<br>**Restrictions associated with COVID-19 vaccination**: Until 31st March 2022, non-pharmaceutical interventions varied regionally according to a weekly risk assessment. Vaccine passports were introduced before the start of the study period, and they were gradually eliminated from 31st March 2022 to 15th June 2022.
**Navarre (Spain)**	**Testing eligibility:** During the study period, the protocol to test children and adolescents did not change. All SARI patients in hospitals were tested by PCR, regardless of the result of rapid antigen tests performed before admission. Swabbing for PCR tests was done in emergency rooms before admission. Tests were free of charge for patients.<br>**Rapid antigen tests:** Tests were free of charge for patients.<br>**Hospital COVID-19 screening practices:** During the study period, the protocol to test children and adolescents did not change.<br>**Restrictions associated with COVID-19 vaccination**:

Supplementary Table S10: Testing policies in place during the study period in each study site (cont.)

Study sites	Testing policies
**Norway**	**Testing eligibility:** COVID-19 PCR tests were freely available for anyone during the study period, and until February 2022, PCR tests were mandatory for respiratory symptoms/positive self-tests.<br>**Rapid antigen tests:** Self-testing was freely available from August 2021 for all families with children attending school. Later in the year, it was available (for free) for everyone. There was also extensive testing among school adolescents with a "test to stay" policy to avoid quarantine among contacts. This means there was much more testing among children/adolescents in this period (August 2021 – December 2021). Schools/municipalities provided tests for children to be tested twice a week. Until February 2022, all positive self-tests needed to be confirmed by a PCR test. After February 2022, this was no longer the case.<br>**Hospital COVID-19 screening practices:** During the study period, COVID-19 PCR testing in hospitalisations was done for those with respiratory symptoms, and these practices did not change over the study period.<br>**Restrictions associated with COVID-19 vaccination**: Norway did not introduce the need for a negative test for any "social" activities, just for travel.
**Portugal**	**Testing eligibility:** During the study, the entire population was eligible to be tested by PCR free of charge. After June 2022, tests were only mandatory for those suspected of COVID-19 and negative self-test results.<br>**Rapid antigen tests:** At the beginning of the study period, mass screening was implemented in schools using TRAG or TAAN. During the study period, these tests were available at pharmacies (not free of charge).<br>**Hospital COVID-19 screening practices:** Before admission, symptomatic individuals were tested with TRAG or TAAN. Negative cases, with continued suspected COVID-19, were PCR tested after 24h.<br>**Restrictions associated with COVID-19 vaccination**: Vaccine passports were introduced before the start of the study period and later on gradually eliminated.

Supplementary Table S11: COVID-19 hospitalisation hazard rate between vaccinated and unvaccinated without a previous SARS-CoV-2 infection, crude and fully adjusted for confounding, for children aged 5-11 by study site, within the VEBIS EHR network

	Denmark	Italy	Navarre (Spain)	Norway	Portugal
**HR Vaccinated/unvaccinated (crude)**	0.13 (0.04 to 0.43)	0.60 (0.53 to 0.67)	0.24 (0.05 to 1.17)	NA	0.56 (0.32 to 0.97)
**HR Vaccinated/unvaccinated (fully adjusted)**	0.13 (0.04 to 0.44)	0.64 (0.56 to 0.72)	0.24 (0.05 to 1.17)	NA	0.53 (0.30 to 0.93)
**Ratio HR_Crude/HR_fully**	1.00	0.94	1.00	NA	1.06

NA – Non-applicable

Supplementary Table S12: COVID-19 hospitalisation hazard rate between vaccinated and unvaccinated without a previous SARS-CoV-2 infection, crude and fully adjusted for confounding, for adolescents aged 12-17 by study site, within the VEBIS EHR network

	Denmark	Italy	Navarre (Spain)	Norway	Portugal
**HR Vaccinated/unvaccinated (crude)**	0.16 (0.09 to 0.29)	0.59 (0.53 to 0.66)	0.08 (0.01 to 0.41)	NA	0.43 (0.26 to 0.71)
**HR Vaccinated/unvaccinated (fully adjusted)**	0.17 (0.10 to 0.31)	0.54 (0.48 to 0.61)	0.10 (0.02 to 0.56)	NA	0.42 (0.25 to 0.69)
**Ratio HR_Crude/HR_fully**	1.07	1.09	0.80	NA	1.02

NA – Non-applicable
